# Let-7b expression determines response to chemotherapy through the regulation of Cyclin D1 in Glioblastoma

**DOI:** 10.1186/1756-9966-32-41

**Published:** 2013-06-27

**Authors:** Yong Guo, Kuipo Yan, Jiasheng Fang, Qiang Qu, Ming Zhou, Fenghua Chen

**Affiliations:** 1 Department of Neurosurgery, Xiangya Hospital, Central South University, 410008, Changsha, China; 2 Department of Cardiology, The First Affiliated Hospital of Henan College of TCM, Zhengzhou 450008, P.R. China; 3 Institute of Clinical Pharmacology, Central South University, 410008, Changsha, China; 4 Cancer Institute, Central South University, 410008, Changsha, China

**Keywords:** Glioblastoma, Let-7b, Cisplatin, Drug resistance, Cyclin D1

## Abstract

**Background:**

Glioblastoma is the most common type of primary brain tumors. Cisplatin is a commonly used chemotherapeutic agent for Glioblastoma patients. Despite a consistent rate of initial responses, cisplatin treatment often develops chemoresistance, leading to therapeutic failure. Cellular resistance to cisplatin is of great concern and understanding the molecular mechanisms is an utter need.

**Methods:**

Glioblastoma cell line U251 cells were exposed to increasing doses of cisplatin for 6 months to establish cisplatin-resistant cell line U251R. The differential miRNA expression profiles in U251 and U251R cell lines were identified by microarray analysis and confirmed by Q-PCR. MiRNA mimics were transfected into U251R cells, and cellular response to cisplatin-induced apoptosis and cell cycle distribution were examined by FACS analysis.

**Results:**

U251R cells showed 3.1-fold increase in cisplatin resistance compared to its parental U251 cells. Microarray analysis identified Let-7b and other miRNAs significantly down-regulated in U251R cells compared to U251 cells. Transfection of Let-7b mimics greatly re-sensitized U251R cells to cisplatin, while transfection of other miRNAs has no effect or slightly effect. Cyclin D1 is predicted as a target of Let-7b through bioinformatics analysis. Over-expression of Let-7b mimics suppressed cyclin D1 protein expression and inhibited cyclin D1-3’-UTR luciferase activity. Knockdown of cyclin D1 expression significantly increased cisplatin-induced G1 arrest and apoptosis.

**Conclusions:**

Collectively, our results indicated that cisplatin treatment leads to Let-7b suppression, which in turn up-regulates cyclin D1 expression. Let-7b may serve as a marker of cisplatin resistance, and can enhance the therapeutic benefit of cisplatin in glioblastoma cells.

## Introduction

Glioma is the first commonly diagnosed types of intracranial tumors, accounting for more than 50% among all primary brain tumors [1]. Gliomas can be classified as astrocytomas, oligodendrogliomas, or tumors with morphological features of both two types of tumors above. According to their degrees of malignancy, gliomas are classified from graded I to IV. Glioblastoma, one subtype of aggressive gliomas, is the most common and lethal brain tumor, with widespread invasion in brain, poor differentiation, destruction of normal brain tissue, and resistance to traditional therapeutic approaches [1-3].

Current options for treatment of glioblastoma include surgical resection of the primary tumor to reduce the tumor size, followed by radiotherapy and adjuvant chemotherapy with temozolomide (TMZ) [4]. However, even with successful surgical resection and subsequent radiotherapy and chemotherapy, the prognosis remains poor, with a median survival of 12–15 months [5]. High tumor recurrence rate and mortality of patients is due to incomplete removal of primary tumors after surgery and resistance to chemotherapy. The infiltrating characteristics of glioblastoma make complete removal of primary tumor virtually impossible, and even cause normal brain tissue damage. Therefore, the limitation of current options for glioblastoma treatment suggests that it is urgently required to study mechanism of chemoresistance regulation of this cancer.

MicroRNAs (miRNAs), a class of 22-nucleotide small non-coding RNAs, can regulate gene expression at post-transcriptional level. MiRNAs are evolutionarily conserved and negatively regulate gene expression. They are transcribed by RNA polymerase II, spliced, and then poly-adenylated to generate primitive miRNAs (pri-miRNAs) [6]. The stem-loop structure of pri-miRNAs can be recognized and cleaved by the nuclear RNase III Drosha to generate hairpin precursor miRNAs (pre-miRNAs). Pre-miRNAs are rapidly exported to the cytoplasm by exportin-5, excised by the cytoplasmic RNase III Dicer to generate a 22-nucleotide miRNA duplex: one strand is a mature miRNA, whereas the other strand (miRNA*) is normally unstable and degraded. The mature miRNAs can suppress target gene expression by interaction with complementary sequences in the 3′-untranslated regions (3′-UTRs) of target mRNAs and trigger translation blockade or mRNA degradation depending on whether it is completely or partially matched with the target genes [7]. Multiple studies have shown that miRNAs are deregulated in various types of human cancers [8], including glioblastoma [9-11], breast cancer [12], lung cancer [13], colon cancer [14], and ovarian cancer [15]. MiRNAs may function as oncogenes or tumor suppressors, and also involve in chemoresistance [15,16].

Cisplatin has dramatically been used as the first line treatment for several types of solid tumors, such as breast, head and neck, ovarian, and lung cancers [17]. Cisplatin in combination with temozolomide has been in clinical trial in malignant glioma patients [18-20]. The combination of temozolomide and cisplatin is safe and effective in the treatment of chemotherapy-naïve GBM patients, and also in pre-treated patients with high-grade glioma refractory to single-agent temozolomide [21,22]. However, cancer cells can develop a resistant phenotype to cisplatin in many patient cases with very poor clinical outcomes [23]. Mechanisms associated with chemoresistance to cisplatin have been investigated, such as up-regulation of drug transporter proteins, aberrancies in DNA damage repair, and apoptosis induction [24]. However, mechanisms of how tumors become resistant to cisplatin have still not been clearly established [25].

To study chemoresistance in glioma, we established a cisplatin-resistant glioblastoma cell line U251R, which is 3.1 fold resistant to cisplatin compared to its parental cell U251. MiRNA expression signature analyzed by microarray identified 16 miRNAs as down-regulated in U251R. Let-7b is one of the most significantly suppressed miRNA. Furthermore, over-expression of Let-7b significantly re-sensitized U251R cells to cisplatin through inhibition of cyclin D1 expression. Cyclin D1 knockdown dramatically increased cisplatin-induced apoptosis and G1 arrest. Taken together, our results suggested that cisplatin treatment leads to Let-7b suppression, which in turn up-regulates cyclin D1 expression, resulting in resistance to cisplatin. Therefore, Let-7b may be considered as a marker for early diagnosis of cisplatin resistance, and restoration of Let-7 in glioblastoma could be a new strategy for cisplatin-resistant cancer treatment in the future.

## Materials and methods

### Reagents, antibodies, and vectors

Fetal bovine serum for cell culture and Lipofectamine 2000 were purchased from Invitrogen (Carlsbad, CA, USA). Anti-β-actin antibody was from Santa Cruz Biotechnology (Santa Cruz, CA, USA). Anti-Bcl-2, Bax, and ppRb antibodies were from Cell Signaling Technology (Danvers, MA, USA). Anti-cyclin D1 antibody was from Abcam (Cambridge, MA, USA). Let-7b mimics expression vector was purchased from Wuhan Genesil Biotechnology (Wuhan, Hubei, China).

### Cell culture

Human neuronal glioblastoma cell line U251 was a gift from Dr. Zhongping Chen (Sun Yat-Sen University, Guangzhou, Guangdong, China). U251 cell line was maintained in Dulbecco’s Modified Eagle’s Medium (Sigma, St. Louis, MO) supplemented with 10% fetal bovine serum (Invitrogen), 100 units/mL penicillin and 100 μg/mL streptomycin (Invitrogen), in a 5% CO2 humidified atmosphere at 37°C.

### Generation of cisplatin-resistant U251 cells in vitro

To generate a cisplatin-resistant cell line, U251 cells were exposed to increasing concentrations of cisplatin. Cisplatin concentrations were increased stepwise from 0.1 μg/mL to 0.5 μg/mL when the cells resumed growth kinetics similar to the untreated parental cells. Cells with the ability to grow in 0.5 μg/mL of cisplatin were obtained 4 months after the initial drug exposure, named as U251R.

### Cell viability

Cell lines were seeded into 96-well plates at a density of 5 × 10^3^ cells/100 μL medium per well. After adherence, cells were treated with various concentrations of cisplatin for 48 h, with DMSO as negative controls. At the end of treatment, the tetrazolium compound, 3-(4,5-dimethylthiazol-2-yl)-2,5-diphenyl tetrazolium bromide (MTT, Sigma) was added and then incubated for additional 4 h at 37°C in the dark. The formazan crystals were dissolved by DMSO, and the absorbance was recorded using an ELISA plate reader.

### Plasmid construction

Cyclin D1 shRNA (cyclin-sh) and negative scramble shRNA (SCR) were inserted into pGPHI vector. The primers were as follows: For cyclin-sh, forward primer 5-CACCGATCGTCGCCACCTGGATGTTCAAGAGACATCCAGGTGGCGACGATCTTTTTTG-3, and reverse primer 5-GATCCAAAAAAGATCGTCGCCACCTGGATGTCTCTTGAACATCCAGGTGGCGACGATC-3; for SCR, forward primer 5-CACCGTTCTCCGAACGTGTCACGTCAAGAGATTACGTGACACGTTCGGAGAATTTTTTG-3, and reverse primer 5-GATCCAAAAAA TTCTCCGAACGTGTCACGTAATCTCTTGACGTGACACGTTCGGAGAAC-3. Cyclin D1 3’-UTR sequence was cloned into pGL3-Luc vector. The primers were as follows: forward primer 5-GCTCTAGAGCTGACTCCAAATCTCAATGAAGCCA-3, and reverse primer 5-GCTCTAGAGCTAACCAGAAATGCACAGACCCAG-3.

### MiRNA microarray analysis

Total RNA was extracted from each cell line using TRIzol reagent (Invitrogen) according to the manufacturer's instructions. The RNA samples were submitted to KangChen Bio-tech (Shanghai, China), then labeled with Hy3™ fluorescent dye for hybridization on a miRCURY™ LNA microRNA array (Exiqon, Vedbaek, Denmark). Expression levels of selected miRNAs differed by at least 2-fold between cisplatin-resistant U251R cell line and parental U251 cell line.

### Immunoblot analysis

Cell lysates were loaded onto 10% SDS–polyacrylamide gels, electrophoresed and transferred to PVDF membranes (Millipore, Billerica, MA, USA). Membranes were blocked in TBS-Tween-20 containing 5% non-fat milk at room temperature for 1 h and then incubated with primary antibodies at 4°C overnight. On the second day, the blots were incubated with HRP-linked secondary antibodies at room temperature for 1 h. After three times’ wash in TBST buffer, the blots were visualized by ECL Reagent (Cell Signaling Technology) as previously described [26].

### Luciferase reporter assay

This assay was performed as previously described [27]. Briefly, cells were seeded in a 24-well plate and transfected with miRNA mimics expression vectors, additional pGL3-Luc/cyclin D1-3’-UTR plasmid, and pRL-TK plasmid. Twenty-four hours after transfection, cells were lysed and then luciferase activities were measured according to the manufacturer’s protocol (Promega, Madison, WI, USA). Each sample’s luciferase activity was normalized to that of renilla.

### MiRNA qRT-PCR detection and quantification

Total RNA was isolated from tissues using Trizol (Invitrogen) following the manufacturer’s instructions. RNA was converted to cDNA with Reverse Transcription System (Promega) according to the manufacturer’s instructions. Q-PCR was performed using the miRNA SYBR Real-time PCR kit (Guangzhou RiboBio, Guangzhou, Guangdong, China) on the ABI 7300 Real-Time PCR system (Life Technologies, Grand Island, NY). To calculate relative expression, the (ΔΔCT) method was used in comparing miRNA expression in U251R cells to U251 parental cancer cells according to ABI’s protocol.

### Annexin V-FITC apoptosis detection

This assay was performed according to the manufacturer’s instructions (Beyotime Institute of Biotechnology, Shanghai, China). Briefly, after treatment, cells were collected, washed with PBS and pelleted. Cell pellets were resuspended in 100 μL of Annexin V-FITC labeling solution and incubated at room temperature in dark for 30 minutes. After incubation, reaction was stopped by adding 300 μL ice-cold PBS and measured on FACSCalibur flow cytometer (Becton Dickinson, Franklin Lakes, NJ).

### Caspase-3 activity analysis

Caspase-3 activity was measured by Caspase-Glo3/7 assay kit (Promega) according to the manufacturer’s instructions.

### Cell cycle analysis

This assay was performed as previously described [28]. Briefly, cells were harvested, washed twice with cold PBS and fixed with 70% cold ethanol overnight. Fixative was discarded and 0.2% Triton X-100 was added to the fixed cells. Cells were washed with PBS again and resuspended in PBS containing 50 mg/mL PI and 1 mg/mL RNase A for 30 min in the dark on ice. The samples were then analyzed on a flow cytometer.

### Statistics

The Student′s t-test was used to compare the difference between two tested groups. A value of *p* < 0.05 was considered as indicating a significant difference.

## Results

### Characterization of the induced cisplatin-resistant U251 cells

**Figure 1:**
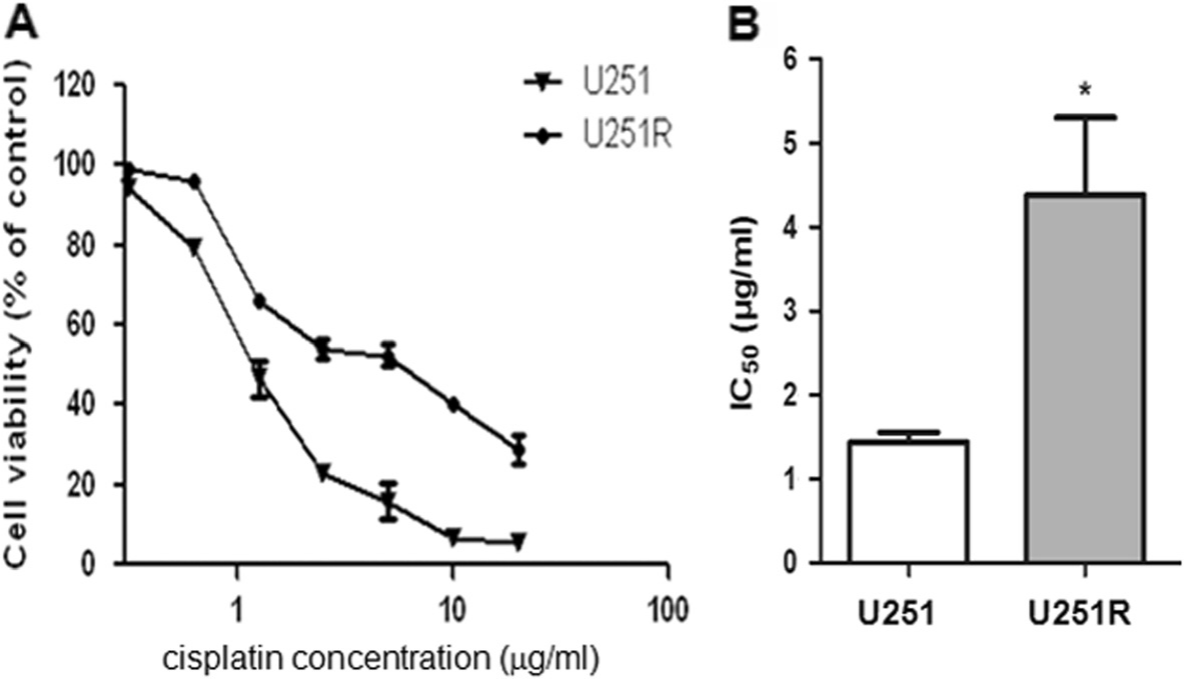
**Characterization of the induced cisplatin**-**resistant U251 cells.** (**A**) U251 and U251R cells were treated with indicated concentration of cisplatin for 72 hours and cell viability was tested by MTT. (**B**) IC_50_ of cisplatin in U251 and U251R cells was calculated.

We observed no apparent difference in morphology or growth rate between the parental U251 cells and cisplatin-resistant U251 cells (hereafter refers as U251R). To compare the sensitivity of the parental U251 and U251R cells to cisplatin, cells were treated with different concentrations of cisplatin for 72 hours and dose–response curves were plotted as shown in Figure 1A. Dose-dependent anti-proliferative activity were observed in both cell lines; however, the resistance of U251R to cisplatin was 3.1 fold higher than that of the parental U251 cells, as measured by the IC_50_ values for cisplatin over 48 hours treatment: 1.4±0.1 μg/mL and 4.4±0.9 μg/mL, respectively (Figure 1B).

### Differential MiRNA expression profiles in U251 and U251R cell lines identified by microarray analysis

**Figure 2:**
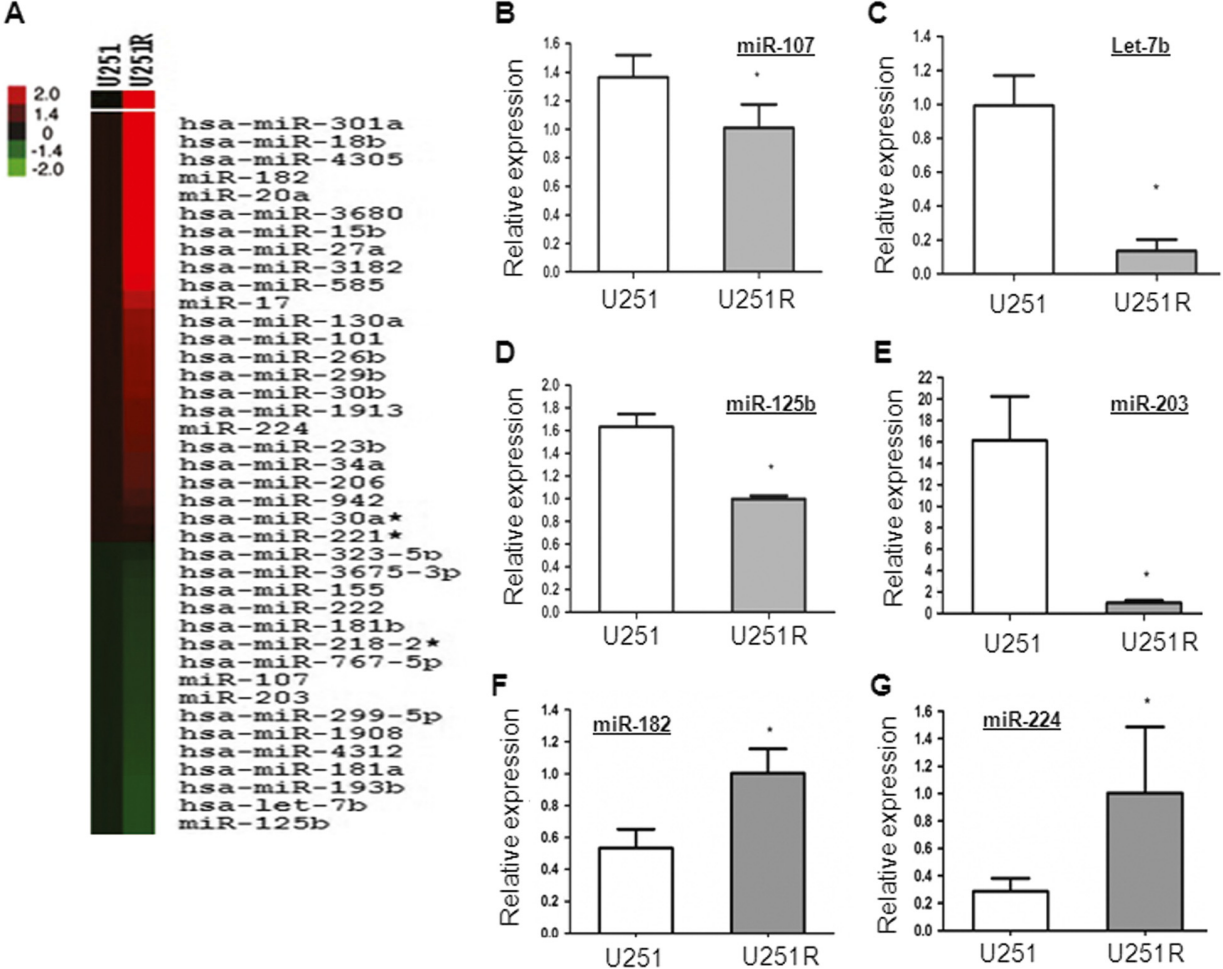
**Differential miRNA expression profiles in U251 and U251R cell lines.** (**A**) MiRNA expression signature was analyzed by miRNA microarray. (**B**-**G**) Selected miRNAs were confirmed by real-time PCR.

Dysregulation of miRNA expression has been reported to be associated with chemoresistance of human cancers. Herein, we performed microRNA microarray containing 3100 probes to analyze differential miRNA expression profiles in U251 and U251R cell lines. As shown in Figure 2A, 23 miRNAs are up-regulated and 16 miRNAs are down-regulated in U251R cells.

The microarray results were then validated by real-time PCR. Consistent with microarray data, miR-182 and miR-224 were up-regulated in U251R cells; Let-7b, miR-125b, miR-107 and miR-203 were significantly suppressed in U251R cells (Figure 2B-G).

### Re-sensitization of the resistant cells by transfection of Let-7b

**Figure 3:**
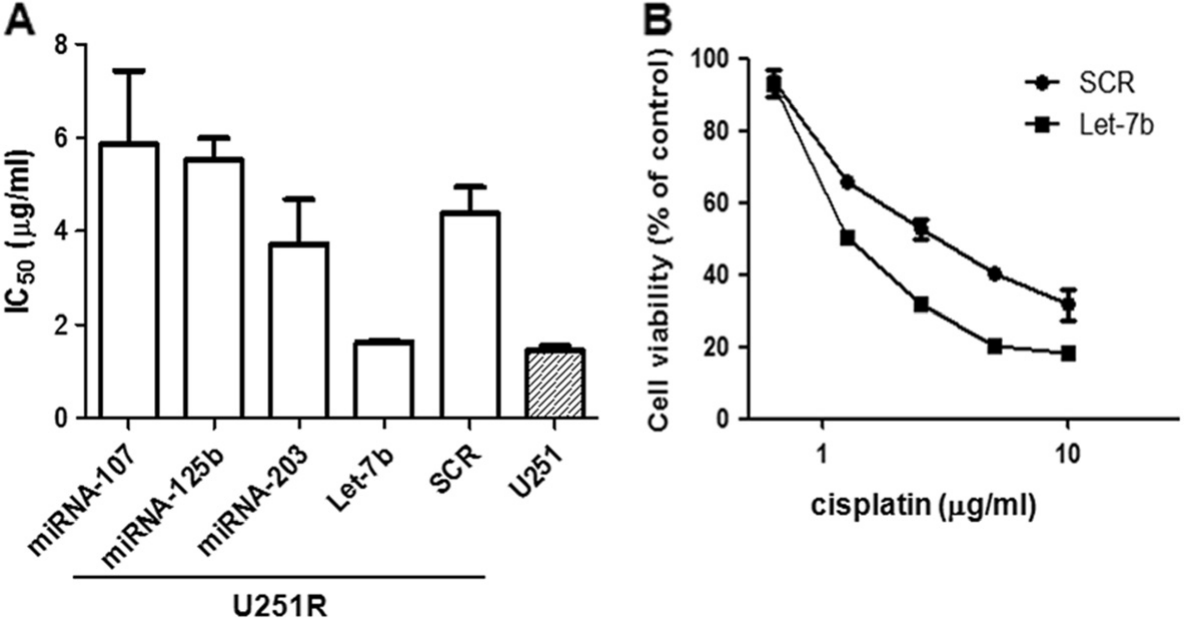
**Transfection of Let-7b re-sensitization of the resistant cells.** (**A**) U251R cells were transfected with mimics of miR-107, miR-125b, miR-203, Let-7b or scramble (SCR). Then their IC_50_ to cisplatin was determined. U251 parental cells were used as control. (**B**) U251R cells were transfected with Let-7b mimics or scramble (SCR), and then the dose–response curves were plotted.

To investigate whether down-regulation of these miRNAs in U251R cells involved in cisplatin resistance, miRNA mimics were transfected into U251R cells, and then their IC_50_ to cisplatin was determined. Interestingly, compared with negative control transfection, transfection of Let-7b greatly sensitized U251R cells to cisplatin, with IC_50_ decreased from 4.38±0.56 μg/mL to 1.62±0.03 μg/mL, which is similar to that of U251 parental cells (1.44±0.11 μg/mL) (Figure 3A). Notably, transfection of neither miR-125b mimics nor miR-107 mimics has significant effect on the sensitivity of U251R cells to cisplatin. MiR-203 mimics lead to moderate inhibition of cisplatin sensitivity. The dose response curves of U251R cells transfected with Let-7b mimics or Scramble to cisplatin were shown in Figure 3B*.* These results suggested that Let-7b plays a critical role in cisplatin resistance, and transfection of Let-7b re-sensitized the U251R cells to cisplatin.

### Transfection of Let-7b increased cisplatin-induced G1 arrest and apoptosis in U251R cells

**Figure 4:**
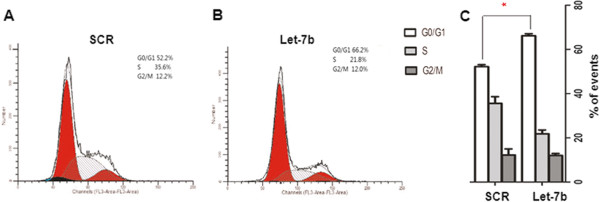
**Let-7b increased cisplatin induced G0/G1 arrest.** U251R cells were transfected with scramble (SCR) (**A**) or Let-7b mimics (**B**) and then treated with cisplatin; cell cycle was detected by flow cytometry. The percentage of cells in different cell cycle phases was calculated (**C**). Data is presented from three independent experiments, and the symbol * indicates statistical difference (*p* < 0.05).

To further confirm the role of Let-7b in cisplatin resistance, cell cycle distribution was analyzed by flow cytometry. Compared with negative control, transfection of Let-7b mimics into U251R cells significantly increased cisplatin-induced G1 arrest (Figure 4A-C).

**Figure 5:**
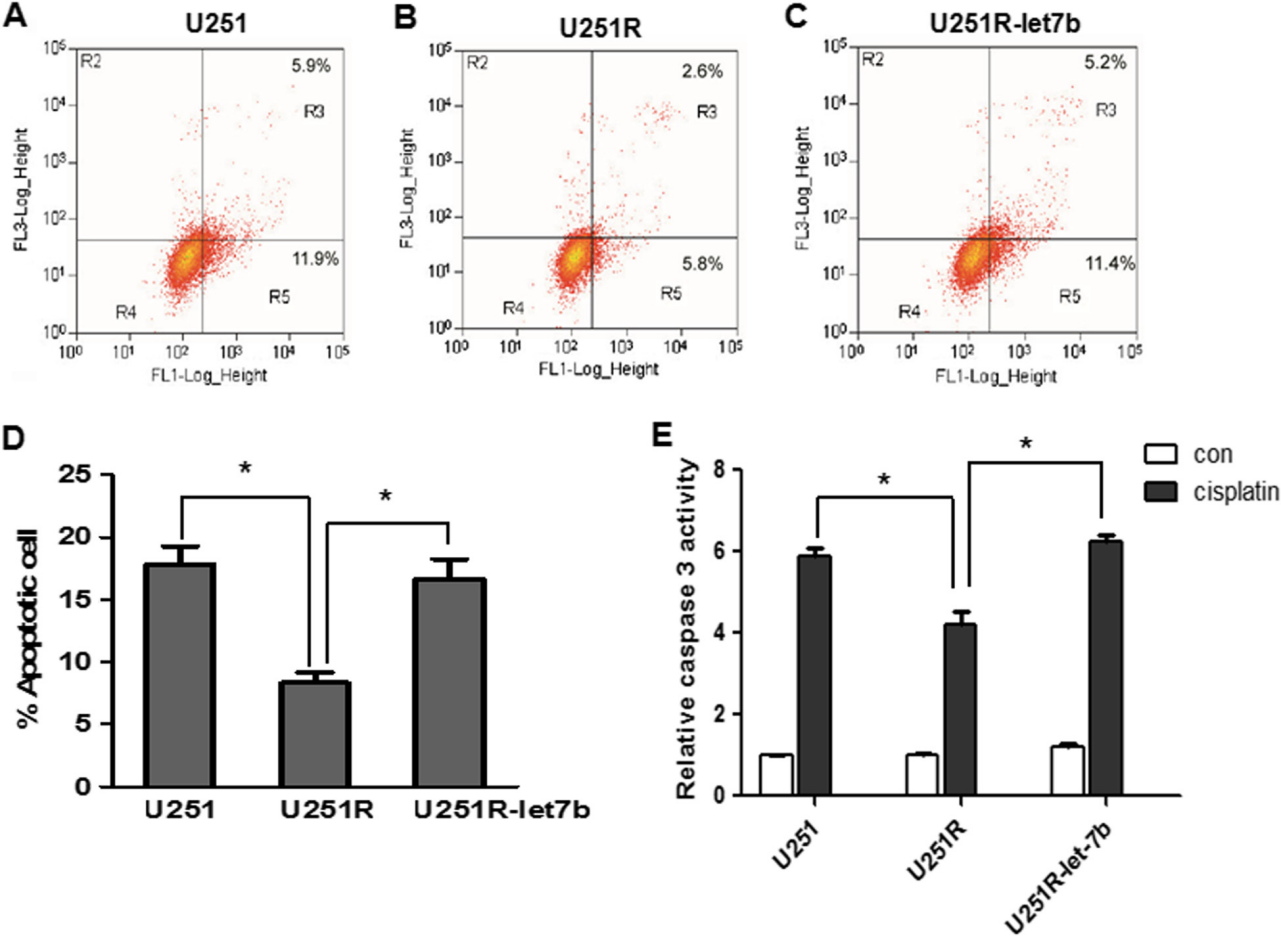
**Transfection of Let-7b increased cisplatin**-**induced apoptosis in U251R cells.** U251 cells (**A**), U251R cells (**B**) or U251R cells transfected with Let-7b (**C**) were treated with cisplatin at 0.625 μg/mL for 48 hours. Cisplatin-induced apoptosis was assessed by Annexin V staining followed by flow cytometry. Right-hand quadrants indicate Annexin V positive cells, indicative of apoptosis. (**D**) The percentage of apoptotic cells was calculated from at least three separate experiments. (**E**) U251, U251R and U251R transfected with Let-7b mimics were treated with cisplatin for 48 hours, and caspase-3 activity was measured. The results were presented as mean±SD (n = 3) (*p < 0.05).

The cisplatin-induced apoptosis was examined by Annexin V/PI staining (Figure 5A-C). Consistently, Let-7b mimics increased cisplatin-induced apoptosis in U251R cells compared with scramble transfection (16.66±1.57% Vs. 8.32±0.85%, *p* < 0.05). Notably, the apoptosis in U251R transfected with Let-7b is comparable to that in U251 parental cells (16.66±1.57% vs. 17.82±1.47%, *p* > 0.05) (Figure 5D).

The caspase-3 activity was determined. After 0.625 μg/mL cisplatin treatment for 48 hours, caspase-3 activity was significantly increased in U251 cells, but less increased in U251R cells. Interestingly, compared with scramble transfection, cisplatin-induced caspase-3 activity in U251R cells was partially enhanced by transfection of Let-7b mimics (3.92±0.08 vs. 6.23±0.30, *p* < 0.05). In fact, the activity of caspase-3 in U251R-Let-7b cells is similar to U251 parental cells (6.23±0.30 vs. 5.9±0.34, *p* > 0.05) (Figure 5E). Taken together, these results suggested that over-expression of Let-7b reversed the resistance to cisplatin in U251R cells.

### Cyclin D1 acts as a downstream target of Let-7b

**Figure 6:**
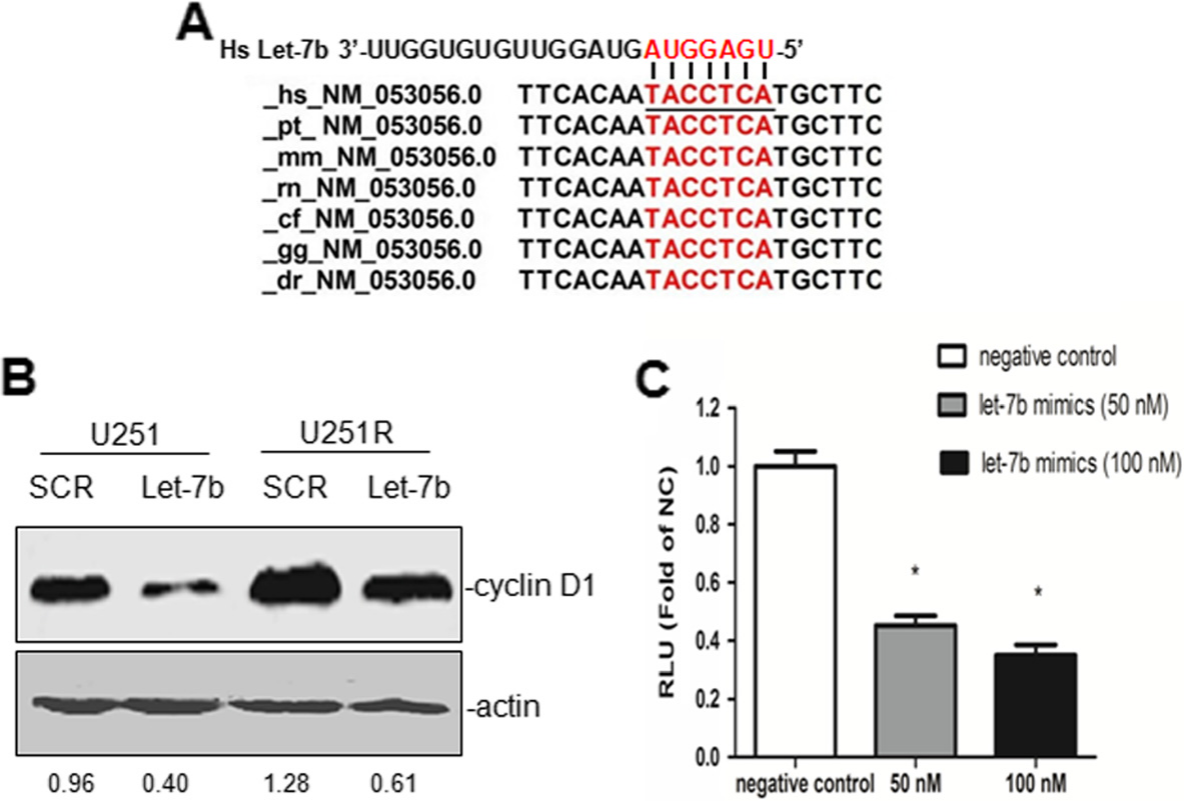
**Let-7b regulated cyclin D1 expression.** (**A**) Prediction of Let-7b binding site in cyclin D1 3’-UTR by TargetScan. (**B**) U251 and U251R cells were transfected with Let-7b mimics or with scramble mimics (SCR). Then cisplatin expression was detected by western blot. (**C**) The cyclin D1-3′-UTR luciferase construct was co-transfected into U251 cells with indicated concentration of Let-7b mimics or with a scramble mimics (SCR) as negative control. Each sample’s luciferase activity was normalized to that of renilla, and results were expressed as mean±SD (n = 3) (*p < 0.05).

To clarify the mechanism of Let-7b-induced changes in chemosensitivity, we first used miRBase and TargetScan to predicted Let-7b target genes, and potential Let-7b binding site is found in 3'-UTR of cyclin D1 (Figure 6A).

To validate if cyclin D1 is a real target of Let-7b, Let-7b mimics was transfected into U251 and U251R cells. As shown in Figure 6B, transfection of Let-7b mimics greatly inhibited cyclin D1 expression both in U251 cells and U251R cells. To test if this is a direct regulation, 3'-UTR of cyclin D1 was cloned into a luciferase expression vector. The data showed that Let-7b mimics inhibited cyclin D1-3’-UTR luciferase activity in a dose-dependent manner (Figure 6C). Overall, these results suggested that Let-7b directly targets cyclin D1 and inhibits cyclin D1 expression.

### Knockdown of cyclin D1 expression increased cisplatin-induced G1 arrest and apoptosis

**Figure 7:**
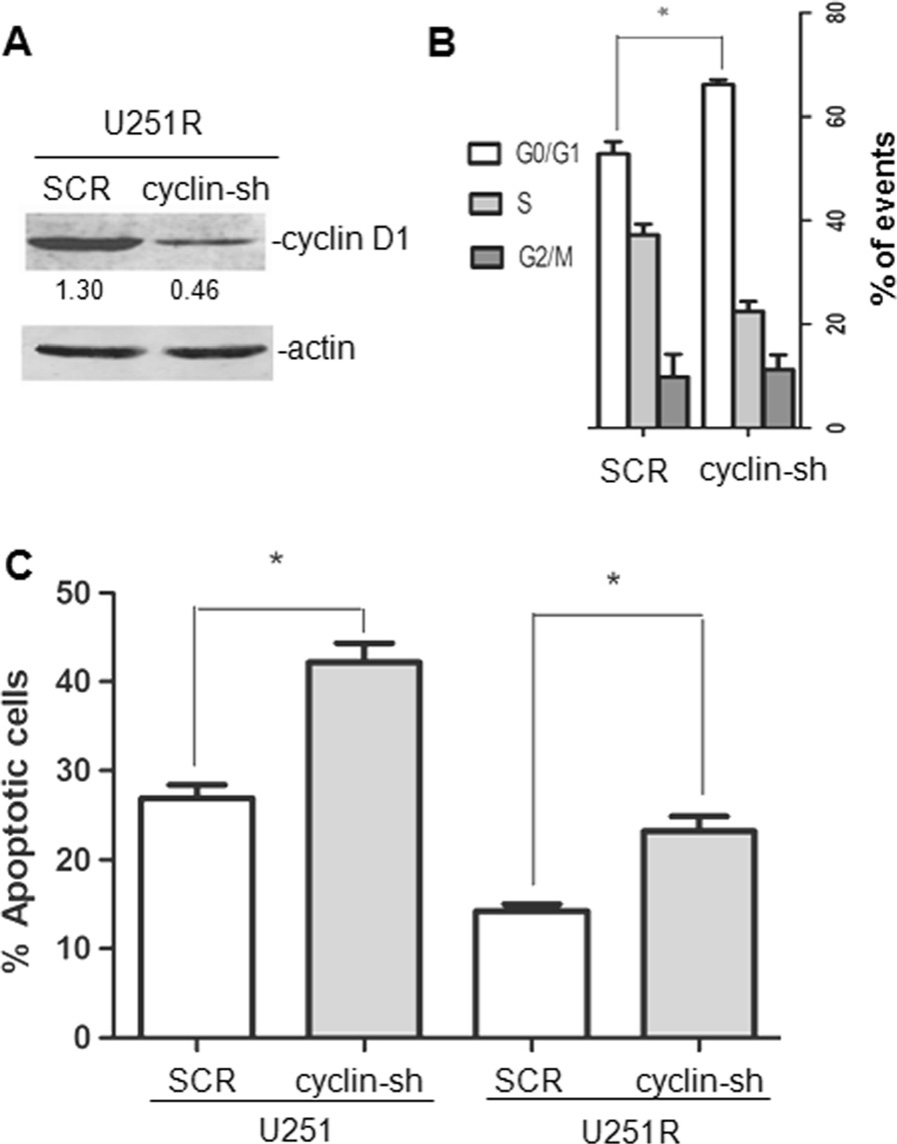
**Knockdown of cyclin D1 expression increased cisplatin induced G1 arrest and apoptosis in U251R cells.** (**A**) U251R cells were transfected with shRNA against cyclin D1 or scramble (SCR), and expression of cyclin D1 was validated by western blot. (**B**) Cells were treated with cisplatin 0.625 μg/mL for 48 hours, then cell cycle was detected by flow cytometry. (**C**) Cisplatin-induced apoptosis was assessed by Annexin V staining followed by flow cytometry.

Amplification, mutation, and high expression of cyclin D1 are reported to be associated with resistance to chemotherapy and poor prognosis in breast tumors, brain tumors and testicular germ cell tumors. To test if cyclin D1 plays an important role in cisplatin resistance in U251R cells, cyclin D1 expression was knockdown by shRNA (Figure 7A). Cisplatin triggered G1 arrest was increased by cyclin D1-shRNA (Figure 7B). Consistently, the apoptosis induced by cisplatin was increased by cyclin D1-shRNA (Figure 7C).

## Discussion

Current anti-cancer chemotherapeutic agents for glioblastoma have not significantly improved the survival of glioblastoma patients during the past ten years [16]. Those patients succumb to their disease mostly for the reason of chemoresistance. Chemoresistance may be either inherent (intrinsic resistance), or induced by chemotherapeutic drugs (acquired resistance) [29]. Intrinsic resistance to anti-cancer drugs results from various factors, including somatic cell genetic diversification in tumors and individual variations of patients. Acquired drug resistance occurs when a tumor that initially sensitive to an anti-cancer drug becomes resistant to that treatment. One prevalent reason for acquisition of chemoresistance is induction of energy-dependent transporter proteins that pump anti-cancer drugs out of cells, and other mechanisms of chemoresistance including resistance to drug-induced apoptosis may also play an important role in acquired drug resistance. Furthermore, recent study indicates that intrinsic and acquired resistances have some similar profiles [30]. So far, there is no effective strategy to overcome chemoresistance. Moreover, drug resistance can only be identified after long-time treatment until now. Therefore, early diagnosis to indicate drug resistance is essential for optimizing therapeutic strategy, avoiding unnecessary treatment and drug-induced side effects. In view of this fact, the research on mechanisms of chemoresistance regulation, the early diagnosis of drug resistance, and the development of novel and effective anti-cancer therapies against glioblastoma are urgently required. In this study, Let-7b down-regulation is associated with acquired cisplatin resistance in U251R cells. Let-7b mimics re-sensitized U251R cells to cisplatin through suppression of cyclin D1 protein expression. Based on these findings, Let-7b might be considered as an early diagnostic marker of cisplatin resistance, and restoration of Let-7b could overcome cisplatin resistance in glioblastoma cells.

Recently, miRNA has been proved as one of the critical regulators during glioma progression. Both up-regulation and down-regulation of miRNAs are involved in the development of glioblastomas and chemoresistance. Shi et al. showed that over-expression of miR-21 could attenuate TMZ-induced apoptosis in U87MG cells through up-regulation of Bax, reduction of Bax/Bcl-2 ratio and caspase-3 activity, demonstrated that miR-21 over-expression is associated with resistance to chemotherapeutic drug TMZ [31]. Furthermore, Li et al. demonstrated that miRNA-21 targets LRRFIP1 which inhibits NF-κB activation. NF-κB pathway is activated upon miR-21 over-expression, exhibits significant anti-apoptotic efficacy, and contributes to VM-26 resistance in glioblastoma [32]. Based on these findings, miR-21 could be a potential target to increase the chemotherapeutic efficacy during glioblastoma treatment. Another study indicated that using an established U251 cell line resistant to temozolomide, Ujifuku et al. performed an analysis of miRNA expression in this cell line and its parental cell line. Three miRNAs miR-195, miR-455-3P, and miR-10a were identified as the most up-regulated miRNAs in the U251 cell line resistant to temozolomide. Knockdown of miR-195 inhibited tumor cell growth, suggesting that it could be a potential target for treatment of glioblastoma with acquired TMZ resistance [33]. In our study, Let-7b was down-regulated in acquired cisplatin-resistant U251R cells. Furthermore, ectopic Let-7b can increase the sensitivity of U251R cells to cisplatin through inhibition of cyclin D1 expression. In this regard, Let-7b could overcome cisplatin resistance in glioblastoma cells, indicating that it could be applied to treat glioblastoma patients with cisplatin resistance.

It is known that Let-7 modulates chemosensitivity in various types of cancer. Let-7 inhibited gemcitabine chemoresistance in pancreatic cancer [34], and could also negatively modulate the chemoresistance in Head and neck cancer [35]. Sugimura et al. showed that Let-7b and Let-7c expression were down-regulated in cisplatin-resistant esophageal cancer cell lines compared with their parent cell lines [36]. Transfection of Let-7 into esophageal cancer cell lines restored their sensitivity to cisplatin. Furthermore, low expression of Let-7b and Let-7c in before-treatment patients is correlated with poor response to cisplatin-based chemotherapy, so Let-7 can also be used as a marker to predict the sensitivity to cisplatin treatment [36]. Moreover, Let-7b down-regulated cyclin D1 expression through targeting 3’-UTR of cyclin D1 mRNA, and inhibited cell cycle progression in melanoma cells [37]. Let-7 also regulates cyclin D1 in other types of tumors. It is reported that Let-7 miRNA inhibited cell growth partially by decreasing mRNA expression of cell cycle stimulators MYC and cyclin D1 in thyroid cancer [38]. Zhao et al. demonstrated that Let-7b regulates neural stem cell proliferation and differentiation by targeting cyclin D1 [39]. Our results also indicated that down-regulation of Let-7b was correlated with cisplatin resistance in glioblastoma cells, and Let-7b could attenuate cyclin D1 expression then dampen chemoresistance of U251R cells to cisplatin. Overall, restoration of Let-7 in glioblastoma may offer a new approach for cancer treatment in the future.

Cyclin D1 belongs to a family of protein kinases that involved in cell cycle regulation. Cyclin D1 has been proved to be associated with chemoresistance to cisplatin-based therapy. Noel et al. demonstrated that cyclin D1 expression was significantly higher in chemoresistant testicular germ tumor cell lines comparing with the parental cells. Furthermore, cyclin D1 knockdown in combination with cisplatin treatment inhibited tumor cell growth more effectively than single treatments [40]. In pancreatic tumor cells, over-expression of cyclin D1 also dramatically reduced chemosensitivity and prolonged survival time upon cisplatin treatment, and knockdown of cyclin D1 resulted in impaired resistance to cisplatin-induced apoptosis [41,42]. Moreover, inhibition of cyclin D1 expression in human pancreatic cancer cells enhances their responsiveness to multiple chemotherapeutic agents other than cisplatin, including 5-fluorouracil, 5-fluoro-2'-deoxyuridine, and mitoxantrone [43]These findings demonstrate that up-regulation of cyclin D1 may be a major reason of cisplatin resistance in multiple tumors. In this regard, cyclin D1 could be a potential marker for treatment evaluation and a candidate target to improve the treatment of cisplatin-resistant tumors. Our study indicated that Let-7b might down-regulate cyclin D1 protein expression through targeting its 3’-UTR. Therefore, cyclin D1 down-regulation induced by restoration of Let-7 in tumors might be a novel therapeutic strategy for cisplatin-resistant glioblastoma treatment.

To sum up, we generated a cisplatin-resistant glioblastoma cell line U251R, and analyzed miRNA expression profiles in U251R compared with its parental cell line U251. Microarray data indicated that Let-7b was dramatically down-regulated in U251R cells compared with U251 cells. Furthermore, ectopic expression of Let-7b remarkably inhibited U251R cell chemoresistance to cisplatin through cyclin D1 expression blockade. Cyclin D1 knockdown significantly promoted cisplatin-induced apoptosis and G1 arrest. In conclusion, Let-7b could be considered as a novel marker of cisplatin resistance during early diagnosis, and more importantly, restoration of Let-7 in tumor cells could offer a novel therapeutic approach for cisplatin-resistant glioblastoma treatment.

## Competing interests

The authors declare no competing financial interests.

## Authors’ contributions

YG, KY, and QQ were involved in the design of the study, performance of the experiments, data analysis, and manuscript writing. JF and MZ participated in the experimental design and data analysis. FC conceived of the study, and was involved in financial support, the design of the study, data analysis, and final approval of the manuscript. All the authors read and approved the manuscript.
